# Chimeric antigen receptor-T cell therapy for T cell-derived hematological malignancies

**DOI:** 10.1186/s40164-024-00584-6

**Published:** 2024-11-28

**Authors:** Haiqiong Zheng, Houli Zhao, Shi Han, Delin Kong, Qiqi Zhang, Mingming Zhang, Yijin Chen, Meng Zhang, Yongxian Hu, He Huang

**Affiliations:** 1 Bone Marrow Transplantation Center of The First Affiliated Hospital & Liangzhu Laboratory, Zhejiang University School of Medicine, Hangzhou, China; 2 Institute of Hematology, Zhejiang University, Hangzhou, China; 3 Zhejiang Province Engineering Research Center for Stem Cell and Immunity Therapy, Hangzhou, China

**Keywords:** CAR-T cell therapy, T cell malignancies, Clinical trials, Fratricide, T cell aplasia, Allogeneic CAR-T cells, Autologous CAR-T cells, HSCT

## Abstract

Relapsed/refractory T cell-derived malignancies present with high heterogeneity and poor prognoses. Recently, chimeric antigen receptor (CAR)-T cell therapy has shown remarkable safety and efficacy in the treatment of B cell-derived malignancies. However, the treatment of CAR-T cells in T cell-derived malignancies has more limitations, such as fratricide, T cell aplasia, and tumor contamination, mainly because of the similarity between normal and malignant T cells. Pan-T antigen CAR-T cells (such as CD5 and CD7 targets), the most widely used CAR-T cells in clinical trials, can cover almost all T cell-derived malignant cells but can also induce severe killing of CAR-T cells and normal T cells. Compared to autologous sources of CAR-T cells, allogeneic CAR-T cells can prevent tumor contamination and become universal products by gene-editing. However, none of these CAR-T cells could completely prevent immune deficiency and disease relapse after T-targeted CAR-T cell therapy. In this review, we summarize the current challenges of CAR-T cell therapy for T cell-derived malignancies in clinical practice and potential strategies to address these limitations.

## Introduction

T cell-derived hematological malignancies constitute a highly heterogeneous group of diseases with a poor prognosis [1]. Based on the maturity of the affected T cells, T cell-derived malignancies can be divided into T precursor-originated malignancies and mature T cell malignancies. T cell acute lymphoblastic leukemia/lymphoma (T-ALL) (15–25% in ALL) and peripheral T cell lymphomas (PTCLs) (15% of non-Hodgkin lymphoma) are the most common types, respectively [1, 2]. Currently, chemotherapy is the first-line treatment for T cell-derived malignancies. Although the complete remission (CR) rate after induction chemotherapy reaches 94% in patients with T-ALL, the 5 year overall survival (OS) rate is only 48%. There were 42% of patients with T-ALL relapse within 5 years of CR. PTCLs are also prone to relapse, with a median time from primary treatment to relapse or progression of 6.7 months [2]. Hematopoietic stem cell transplantation (HSCT) is a potential therapy for relapsed/refractory (R/R) T cell-derived malignancies. However, it can cause profound complications such as graft versus host disease (GvHD), and the OS after HSCT is not satisfactory. The 3 year OS for R/R T-ALL after allo-HSCT was estimated to be 30%, whereas the 5 year OS for PTCLs after auto-HSCT and allo-HSCT was estimated to be 32% and 52%, respectively [2]. Therefore, there is an urgent need to develop novel and effective therapies for this type of malignant disease.

Chimeric antigen receptor (CAR) T-cells are highly effective in treating B cell malignancies [3]. Thus, developing CAR-T cells that target T cell-derived malignancies is a potential strategy. However, CAR-T cell therapy for T cell-derived malignancies is challenging because of the lack of specific malignant T antigens, which may lead to CAR-T cell fratricide, normal T cell aplasia, and malignant T cell contamination during manufacturing and treatment [4]. CAR-targeted antigens can be divided into pan-T antigens and restricted-T antigens, both of which have advantages and disadvantages. Moreover, allogeneic and autologous T cell-derived CAR-T cells present different benefits and drawbacks. This review summarizes the current limitations of novel T-targeted CAR-T cell therapy and the clinical trials of different types of CAR-T cells for T cell-derived malignancies, including their efficacy, side effects, and potential improved strategies.

## Current challenges of CAR-T cell therapy for T cell-derived malignancies

**Fig. 1:**
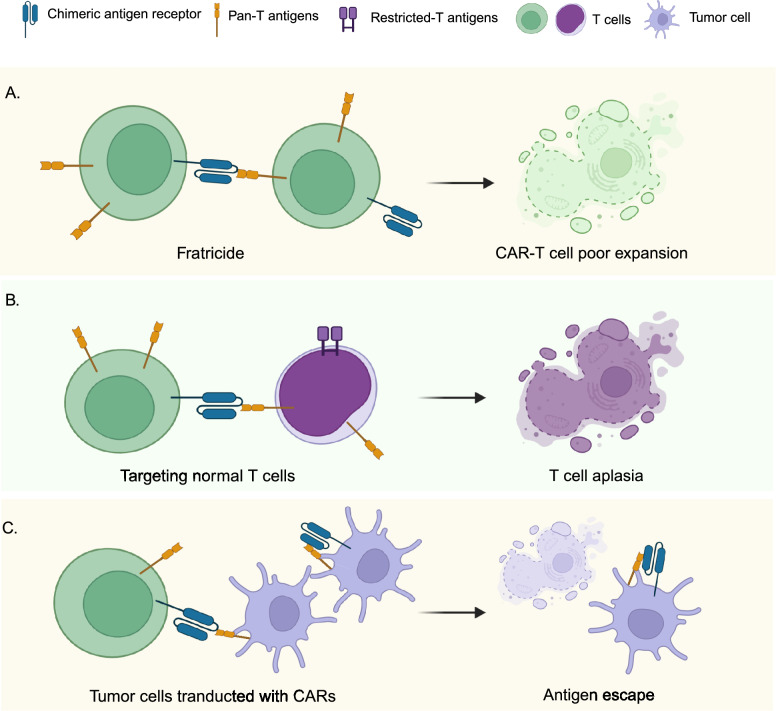
Challenges of CAR-T cell therapy for T cell-derived malignancies. **A:** Fratricide is caused by recognizing target antigens on other CAR-T cells, and can result in the poor expansion of CAR-T cells in vitro culture. **B:** The recognition of target antigens on the surface of normal T cells and subsequent killing can lead to T cell aplasia. **C:** For autologous CAR-T cells, CAR molecules can also be transfected to the contaminated tumor cells, which may cause antigen escape

The ideal CAR-T cell target antigens for T cell-derived malignancies are highly expressed on the surface of all malignant T cells but not on normal tissues. However, T cell-derived malignancies are usually heterogeneous populations, leading to the rare existence of optimal antigens present in all tumor cells [1]. Moreover, if CAR-T cells share antigens with normal T cells, they may recognize and eliminate three types of cells: malignant T cells, other CAR-T cells, and normal T cells (Fig. 1).

The killing among CAR-T cells is also called fratricide and may cause poor expansion, persistence, and antitumor efficacy of CAR-T cells in vivo. T cell aplasia caused by CAR-T cell infusion currently has no effective treatment and may increase life-threatening opportunistic infections in patients [4]. It is entirely possible that malignant T cells could also be collected for CAR transduction because malignant T cells are often present in the peripheral blood of T-ALL patients and some T cell lymphomas (TCLs) patients and share surface antigens with normal T cells. Consequently, CAR-T cells may be contaminated by malignant T cells, which can lead to antigen escape during CAR-T cell therapy [4]. Recently, Hill et al. reported that the CAR-T cell product from a patient with adult T cell leukemia/lymphoma was contaminated with tumor cells, and finally abandoned [5].

In the following sections, we elaborate on the diverse target antigens and T cell sources of CAR-T cells that can potentially resolve the limitations of existing clinical trials.

## Potential target antigens for T cell-derived malignancies

**Fig. 2:**
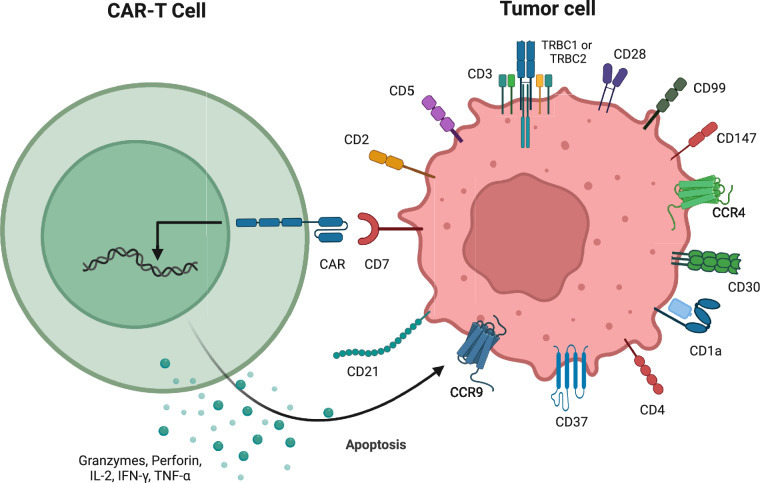
Different target antigens of T cell-derived malignancies for CAR-T cell therapy. CAR-T cells can recognize their target antigens (such as CD5, CD7, CD4, TRBC1) on the surface of tumor cells and then kill tumor cells through secreting granzymes, perforin, IFN-γ, and so on

Target antigens for T cell-derived malignancies can be divided into antigens that cover all T cells and antigens limited to a subset of T cells. CAR-T cells equipped with pan-T antigens can theoretically cover all T cell-derived tumor cells. Still, they may also recognize and eradicate normal T cells, leading to fratricide and T cell aplasia. Antigens limited to a subset of T cells may avoid severe fratricide and T cell aplasia; however, the appropriate tumor types must be carefully screened Fig. 2. The characteristics and efficacy of CAR-T cells for T cell-derived hematological malignancies in clinical trials are summarized in Table 1 [5–15].

**Table 1:** Characteristics and efficacy of CAR-T cells for T cell-derived hematological malignancies in clinical trials

Target antigens	Sources of CAR-T cells	CAR-T cell manufacture	Advantages	Trial phase	Sample size	Efficacy (CR rates)	Toxicities	[References]
CD4	Autologous	CD4-restricted CAR-T cells	Only attacking CD4-positive normal T cells	I	1	100%	CRS	[6]
TRBC1	Autologous	TRBC1-restricted CAR-T cells	Only attacking TRBC1-positive normal T cells	I/II NCT03590574	9	55.6%	CRS, cytopenia	[7]
CD5	Autologous	Natural selection	Without genetic modifications	I NCT0308190	9	22.2%	CRS, ICANS, cytopenia, BK virus and CMV reactivation	[5]
CD7	Autologous	Natural selection	Without genetic modifications; A higher proportion of CD8-positive subset	I NCT04572308	18	94.4%	CRS, ICANS, cytopenia, CMV reactivation	[8]
CD5	Autologous	Natural selection + TKIs	Without genetic modifications; inhibiting strong fratricide and tonic signaling among CAR-T cells	I NCT03081910	4	50%	CRS, cytopenia, EBV reactivation	[9]
CD7	Autologous	Blocking CD7 protein in ER/Golgi	Without genetic modifications; resisting fratricide among CAR-T cells	I NCT04004637	8	75%	CRS, cytopenia	[10]
CD7	HSCT-derived, HLA-matched, and haploidentical donors	Blocking CD7 protein in ER	Without genetic modifications; resisting fratricide among CAR-T cells and preventing tumor pollution during CAR-T preparation	I NCT04689659	20	90%	CRS, ICANS, cytopenia, GvHD, CMV and EBV reactivation	[11]
CD5	HSCT-derived, and new matched donors	CRISPR/Cas9 CD5-KO	Resisting fratricide among CAR-T cells and preventing tumor pollution during CAR-T preparation	I NCT05032599	12	100%	CRS, ICANS, cytopenia, GvHD, EBV reactivation	[12]
CD7	Universal	CRISPR/Cas9 CD7 KO, TCR KO, HLA-II KO and NK cell inhibitory receptor introduced	Resisting fratricide, GvHD, and allo-rejections from CD4-positive T cells and NK cells	I NCT04538599	11	63.6%	CRS, cytopenia, CMV and EBV reactivation	[13]
CD7	Universal	CRISPR/Cas9 CD7 KO and TCR KO	Resisting fratricide and GvHD	I ChiCTR1900025311	12	91.7%	CRS, cytopenia	[14]
CD7	Universal	Base editing CD7 KO, TCR KO, and CD52 KO	Editing more precise and without DNA double strands breaking; resisting lymphodepleting serotherapy, fratricide, and GvHD	I ISRCTN15323014	3	100%	CRS, cytopenia, CMV and EBV reactivation	[15]

Characteristics of CAR-T cells for T cell-derived hematological malignancies in clinical trials, including target antigens, sources of CAR-T cells, CAR-T cell manufacture, advantages, trial phase, sample size, efficacy, toxicities, and references. The efficacy of CAR-T cells is evaluated by CR rate within 1 month

CAR: chimeric antigen receptor; CR: complete response; CRS: cytokine response syndrome; TRBC: T cell receptor beta constant; ICANS: immune effector cell-associated neurotoxicity syndrome; CMV: cytomegalovirus; TKIs: tyrosine kinase inhibitors; EBV: Epstein-Barr virus; ER: endoplasmic reticulum; HSCT: hematopoietic stem cell transplantation; HLA: human leukocyte antigen; GvHD: graft versus host disease; CPISPR: clustered regularly interspaced short palindromic repeats; Cas9: CRISPR-associated protein 9; KO: knockout; TCR: T cell receptor

### Antigens covering all T cells

Pan-T antigens, specifically CD5 and CD7, are the most widely used target antigens for T cell-derived malignancies in current clinical practices [5, 11]. However, antigens covering all tumors are also expressed on almost all normal T cells, rapidly eliminating other CAR-T cells and normal endogenic T cells when killing malignant T cells. Fratricide and on-target off-tumor effects of CAR-T cells may cause limited CAR-T cell persistence and severe T cell aplasia during treatment, eventually affecting the curative effects of CAR-T cells [16].

Since fratricide is caused by the interaction between antigens and CARs, one strategy to avoid fratricide is to reduce the expression of surface target antigens. Knocking out target genes is an effective way to decrease the expression of surface proteins. Several clinical trials have used different gene-editing techniques (such as clustered regularly interspaced short palindromic repeats (CPISPR)/CRISPR-associated protein 9 (Cas9) and base editing) to achieve a target antigen-negative phenotype in CAR-T cells [13, 15]. Retaining target proteins in the endoplasmic reticulum (ER) and/or Golgi apparatus is an alternative way to decrease target antigens on the cell surface. For example, in 2021, Pan et al. demonstrated that a CAR-T cell product possesses a CD7-binding scFv domain and an ER-anchoring domain to retain CD7 proteins in the ER and achieve a surface CD7-negative phenotype [11].

Recently, several studies have reported that CAR-transduced T cells gradually lose their surface target proteins, such as CD5 and CD7, without any artificial intervention of target genes or proteins; thus, fratricide among these CAR-T cells is partial and transient [5, 8]. Researchers named this phenomenon as “natural selection”. Specifically, they have found that CD5 or CD7 CAR-transduced T cells downregulated their surface target antigens when detected by flow cytometry (FCM), but mRNA and protein were still detectable through quantitative polymerase chain reaction (qPCR) and western blotting [8, 17]. Continuous downregulation of antigens on the cell surface may be caused by antigen internalization, antigen masking, or intracellular sequestration [17]. The decreased surface proteins permit CD5 or CD7 CAR-expressing T cells to resist fratricide, but this belated resistance to fratricide still affects the expansion of CAR-T cells in vitro [8]. Recently, researchers found that tyrosine kinase inhibitors (TKIs) like ibrutinib and dasatinib could minimize fratricide in CAR-T cells (including CD5 and CD7) during natural selection period. The TKI-pretreated CAR-T cells showed good persistence and cytotoxicity both in vitro and in vivo [9, 18]. In a clinical study (NCT03081910), TKI-pretreated CD5 CAR-T cells showed more sustained persistence in patients, and four of seven patients with R/R T-ALL achieved minimal residual disease (MRD)-negative CR [9].

Moreover, recombinant antibodies and the TET-OFF expression system can also be beneficial in avoiding fratricide during the manufacturing of pan-T targeted CAR-T cells. The CD7 recombinant antibody, PUT664, which can block the CD7 antigens on CAR-T cells, prevents fratricide during the in vitro expansion of CD7 CAR-T cells. The TET-OFF expression system is a system that can temporarily regulate the CAR gene and thereby adjust CAR-T cells in a reversible manner. For instance, Mamonkin et al. designed a regulated expression system that reversibly inhibited CAR expression through doxycycline exposure in vitro and restored the CAR expression and antitumor function of transduced T cells in vivo by removing doxycycline before infusion [19].

Possible solutions to T cell aplasia include transient CAR expression technologies (such as mRNA delivery and adeno-associated viral transfection) and safety switches (such as suicide switches, receptor switches, and adapter switches). The transient CAR expression technologies do not integrate vectors into the genome, so that it can avoid potential genotoxicity. The transient CAR-T cells can alleviate T cell aplasia, but the persistence and efficacy in patients remain to be determined. The safety switches can control the duration of CAR-T cells in patients, so that the toxic side effects of CAR-T cells, including T cell aplasia, can theoretically be controlled [19].

In general, there are several emerging technologies for avoiding fratricide and T cell aplasia in developing CAR-T cells with pan-T targets, such as gene-editing, protein-retaining, transient CAR expression technologies, and safety switches. But comprehensive clinical trials are needed to assess the feasibility and safety of each technology in patients.

### Antigens limited to a subset of T cells

Given the severe fratricide and T cell aplasia caused by pan-T targeted CAR-T cells, the restricted-T antigens open a new avenue for CAR-T cell therapy to treat T cell-derived malignancies.

CD4 is an antigen normally expressed in a group of T cells and some T cell-derived malignancies, including most mature TCLs and some T-ALL. In 2019, Zhang et al. reported the first human clinical trial involving CD4 CAR-T cells. A patient with refractory Sézary syndrome who was resistant to chemotherapy presented CAR-T cell expansion and achieved CR on day 13 after infusion [6]. However, CD4 CAR-T cells can retain fratricide skills for peripheral CD4-positive T cells, which may cause CD4-positive T cell aplasia and increase the risk of opportunistic infections.

Another promising target is T cell receptor beta constant 1 (TRBC1) and TRBC2. Although malignant T cells show significantly decreased T cell receptor (TCR) expression, almost all PTCL and 30%-50% of T-ALL cells still express the TCR. Normal T cells are generally a mixture of TRBC1 and TRBC2 T cells, while malignant T cells express only TRBC1 or TRBC2 on their surface, which enables TRBC1 or TRBC2 CAR-T cells to eliminate all relevant malignant T cells and only a subset of normal T cells [16, 20]. In an ongoing phase I/II clinical trial (NCT03590574), AUTO4, a TRBC1-targeted autologous CAR-T cell, showed good antitumor activity and safe prolife in selected patients with TRBC1-expressed PTCL. Among the nine patients evaluated, five achieved a complete metabolic response by positron-emission tomography-computed tomography, and one achieved partial remission [7]. Unfortunately, only two patients remained CR at or over one year, and CAR-T cells were not detectable in all patients. The activity of TRBC1-targeted CAR-T cells might be limited by normal TRBC1-positive T cells, which will impact the persistence and efficacy of CAR-T cells in patients [20].

In addition to CD4 and TRBC1/TRBC2, there are some other restricted-T antigens for CAR-T cell therapy having been on the way to clinical trials, such as CD1a (NCT05745181, NCT05679895), CD30 (NCT01316146, NCT02259556), CD37 (NCT04136275), CCR4 (NCT03602157), and so on. Each restricted-T target can partially overcome fratricide and T cell aplasia; however, at present, there are few studies on CAR-T cells with restrictive antigens. We expect more preclinical and clinical studies to focus on this direction and make breakthroughs in the future to bring patients an alternative choice.

## Autologous and allogeneic CAR-T cells for T cell-derived malignancies

Another category of CAR-T cells includes autologous and allogeneic CAR-T cells. Autologous T cells have no GvHD or rejection, but tumor contamination is a major problem for this kind of CAR-T cells. In contrast, allogeneic CAR-T cells are of high purity and quality, but allogeneic adoptive infusion has limitations, such as GvHD and allo-rejection [4].

### Autologous CAR-T cells

Autologous CAR-T cells have been widely used in clinical trials, have shown good efficacy, and can theoretically persist in vivo for a long time to prevent disease recurrence [4].

In 2022, Lu et al. reported naturally selected CD7 CAR-T cells (NS7CAR) without gene-editing, as described in the previous section. In a phase I clinical trial (NCT04572308), 17 of 18 patients with R/R T-ALL or T cell lymphoblastic lymphoma (T-LBL) achieved MRD-negative CR by day 28 after receiving autologous NS7CAR. Two patients received allo-HSCT donor-derived NS7CAR because of lymphopenia and both achieved MRD-negative CR. And no malignant cell contamination was detected in NS7CAR by FCM or qPCR [8]. Recently, Hill et al. reported a phase I clinical trial (NCT0308190) of autologous unedited CD5 CAR-T cells for treating R/R mature TCLs. Among 17 enrolled patients, only nine received infusion finally; the overall response rate was 44% (four of nine patients). One patient's CAR-T cell product was contaminated with malignant cells and eventually abandoned [5]. Naturally selected autologous CAR-T cells can resist fratricide without additional genetic editing. The selected CD7-negative CAR-T cells had a higher proportion of the CD8-positive central memory subset than the CD7 knockout (KO) or naturally existing CD7-negative T cell-derived CAR-T cells, which rendered these naturally selected and fratricide-resistant CAR-T cells with a better-sustained killing capability [8]. In the manufacturing of naturally selected CAR-T cells, the proportion of CAR was more than 90%, but the number of CAR-T cells was significantly decreased resulting from fratricide. The reduced number of CAR-T cells in natural selection period limits clinical application, but can be improved with TKIs [9, 18].

Zhang et al. successfully produced a CD7-redirected and fratricide-resistant CAR-T cell product by combining a tandem CD7 nanobody with an ER/Golgi-retention signal peptide. Autologous CD7 CAR-T cells effectively removed tumor cells from patients in an open-label phase I clinical trial (NCT04004637). Among the eight enrolled patients with R/R T-ALL/LBL, six (75%) achieved CR within 1 month post-infusion, which increased to seven (87.5%) after 3 months. No tumor contamination was observed during CAR-T product manufacturing in this study [10].

Autologous CAR-T cell therapy is a promising treatment for T cell-derived malignancies, but the challenges it faces cannot be ignored. On the one hand, one case of tumor contamination was observed in CAR-T cell manufacturing, it was mainly due to the similar antigen profiles between tumor cells and normal T cells [5]. Thus, pre-screening all enrolled patients’ immunophenotypes of tumor cells to identify the differences between healthy and malignant T cells can help scientists determine the sorting method and greatly avoid the contamination of tumor cells in CAR-T cell products. On the other hand, some patients experienced failure of CAR-T cell manufacturing or died during CAR-T cell manufacturing [5]. Therefore, in these cases, using allogeneic T cells for CAR-T cell manufacturing may be a suitable method.

### Allogeneic CAR-T cells

Allogeneic T cells have some advantages that autologous CAR-T cells do not have, such as quality- and quantity-stable T cell sources and no tumor contamination [4]. In general, allogeneic CAR-T cells can be divided into four types: HSCT-donor cells (cells from previous HSCT donors), human leukocyte antigen (HLA)-matched donor cells (cells from HLA-matched donors), haploidentical donor cells (cells from haploidentical donors), and universal cells (cells from unrelated healthy donors).

In 2021, Pan et al. demonstrated a CAR-T cell product that could retain de novo-synthesized CD7 proteins in the ER, thus minimizing the expression of surface CD7. These donor-derived fratricide-resistant CD7 CAR-T cells showed efficient antitumor activity in patients with R/R T-ALL, and 90% (n = 18) of patients achieved CR in their phase I clinical trial (NCT04689659). Specifically, at a median follow-up time of 6.3 months, six of seven patients who received CAR-T cells from new donors (including HLA-matched and haploidentical donors) remained remission after subsequent HSCT, and nine of 11 patients from previous HSCT donors remained remission without other treatment [11]. The reasons for efficient proliferation and persistence of these donor-derived CAR-T cells may be discussed in two aspects. Previous HSCT-induced complete chimerism may have contributed to the low rejection rate for those who received prior HSCT donor-derived CAR-T cells. For patients who received new donor-derived CAR-T cells, both pre-lymphodepletion and CAR-T cell-mediated T cell aplasia were possible causes [11].

**Fig. 3:**
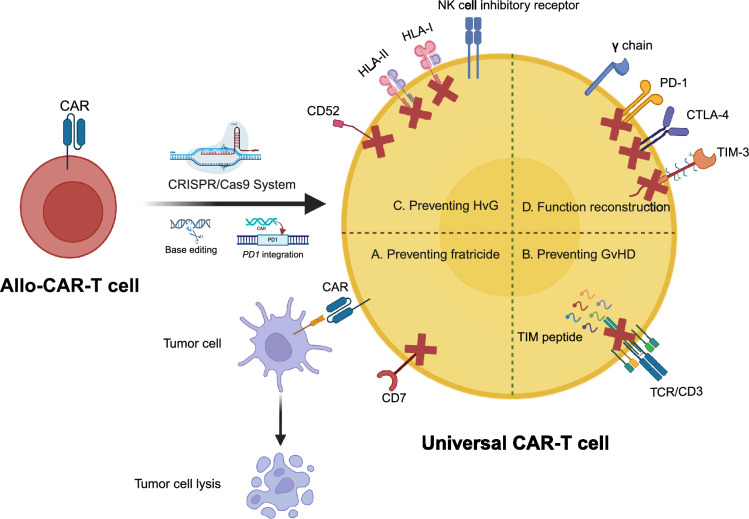
Strategies for universal CAR-T cell manufacturing. A: Knocking out target antigens (such as CD7) through CRISPR/Cas9 or base editing in CAR-T cells can prevent fratricide maximally. B, C: Knocking out TCR/CD3 may alleviate GvHD, while knocking out HLA molecules or CD52 may alleviate HvG. D: To enhance function of CAR-T cells, knocking out inhibitory molecules (like PD-1, CTLA-4, and TIM-3) may be useful

The aforementioned donor-derived CAR-T cells may solve the problem of tumor contamination. However, these healthy donor-derived CAR-T cells need to be personalized, depending on HLA matching. In addition, HSCT donor-derived, HLA-matched or haploidentical donor-derived T cells often require high costs and a long time to be manufactured into CAR-T cells, which causes more patients to miss the best time for treatment. In this regard, our group developed unrelated healthy donor-derived CD7 CAR-T cells (RD13-01) using the CRISPR/Cas9 system, with the genetic modification of CD7 KO to resist fratricide, TCR KO to resist GvHD, HLA-II KO and NK cell inhibitory receptor (extracellular and transmembrane domains of E-cadherin and CD28 intracellular domain) introduced to diminish allo-rejections from CD4-positive T cells and NK cells, respectively. E-cadherin can negatively regulate the function of NK cells by binding KLRG1. Moreover, we introduced an γ chain to rescue the IL-2 deficiency and enhance CAR-T cell proliferation. This universal CAR-T cell product, derived from unrelated healthy donors, is resistant to GvHD and allo-rejection and could theoretically be used in all patients (Fig. 3). In a phase I clinical trial (NCT04538599), seven of 11 patients achieved CR within 28 days, including a patient with acute myeloid leukemia (AML). The median duration of RD13-01 persistence in nine detected patients was 28 (10–120) days. However, we found that most recovered T cells were CD8-positive and displayed allo-reactivity against CAR-T cell donor-derived naïve T cells. As RD13-01 has little allo-reactivity to CD4-positive T cells, these CD8-positive T cells may still play a role in allo-rejection and limit RD13-01 persistence in patients [13].

Another CD7-redirected universal CAR-T product (GC027) comprises a CD7 CAR and an enhancer called C7R. CD7 and TCR were disrupted using the CRISPR/Cas9 system to prevent fratricides and GvHD, respectively, while C7R strongly promotes CAR-T cell expansion via the IL7Ra intracellular segment. In a clinical trial (ChiCTR1900025311), 11 of 12 patients with R/R T-ALL or T-LBL achieved CR after GC027 infusion. Universal GC027 could be detected by FCM and qPCR within 1 month, which was similar to our clinical data. However, CAR-T cells' short persistence (4 weeks) was sufficient for most patients to achieve CR in both studies [13, 14].

Although the two aforementioned studies did not show an off-target effect of the gene-editing on tumorigenesis, the risk was still present in the CRISPR/Cas9 system. Base editing does not rely on double-stranded DNA breakage; therefore, it is considered a safer editing tool than the CRISPR/Cas9 system. Recently, a study reported that CD52, CD7, and TCR beta chains were successfully inactivated using a base editing technique. Thus, the universal CD7 CAR-T cell product (BE-CAR7) could resist lymphodepletion by alemtuzumab, fratricide, and GvHD. In their phase I clinical study (ISRCTN15323014), three patients with relapsed T-ALL who received BE-CAR7 showed morphological remission within 28 days [15].

Related donor-derived CAR-T cells are still dependent on HSCT-derived, HLA-matched, or haploidentical donors. Genome-edited universal CAR-T cells can reduce complications such as GvHD and allo-rejection and become the true “off-the-shelf” products for patients. As a key technology for the manufacturing of universal CAR-T cells, gene-editing technology includes CRISPR/Cas9, base editing, zinc finger nuclease (ZFN) and transcription activator-like effector nuclease (TALEN), and so on. CRISPR/Cas9, ZFN, and TALEN techniques edit genes through double-stranded DNA breakage, causing unpredictable genotoxicity on cells. While base editing edits genes through point mutation, ensuring the efficiency of gene-editing with higher safety [19]. Clinical safety and efficacy are the best indicators to test these CAR-T cells, which will also help us optimize the standard allogeneic as well as universal CAR-T cell manufacturing in the future.

### Comparison of autologous and allogeneic CAR-T cells

Recently, a phase I clinical trial (NCT04823091) compared the efficacy of autologous and allogeneic CD7 CAR-T cell therapy and found that allogeneic CAR-T cells achieved better remission and less disease relapse than autologous CAR-T cells [21]. However, Pan et al. conducted a retrospective study and demonstrated comparable remission rates between autologous and allogeneic CAR-T cells. Thus, autologous CAR-T cells may be more recommended for patients with low tumor load, for the no GvHD and less toxicities [22]. More clinical studies are needed to analyze the advantages and disadvantages of autologous and allogeneic CAR-T cells, in order to help clinicians better choose autologous or allogeneic sources in different clinical situations in the future.

## Complications after CAR-T cell therapy for T cell-derived malignancies

Non-specific complications after CAR-T cell therapy for T cell-derived malignancies such as cytokine response syndrome (CRS), immune effector cell-associated neurotoxicity syndrome (ICANS), GvHD, and cytopenia are shared among B cell lineage target antigens (such as CD19 and BCMA) of CAR-T cells. In recently reported clinical trials for CD7 CAR-T cells, the rates of severe advert events (grade ≥ 3) within 1 month post-infusion were as follows: CRS (0%-66.7%), ICANS (0%-14.3%), GvHD (0%-14.3%), anemia (0%-91.7%), neutropenia (85.7%-100%), and thrombocytopenia (57.1%-95%) [8, 10, 11, 13, 14, 23]. At present, there is nothing special for the non-specific complication management of T-targeted CAR-T cell therapy. Strategies to mitigate the occurrence of serious CRS and/or ICANS encompass administering low doses of CAR-T cells to patients with a high tumor burden and modifying the functional structure of CAR-T cells, since high tumor burden and CD28 costimulatory domain are risk factors associated with CAR-T cell-related toxicities [24].

**Fig. 4:**
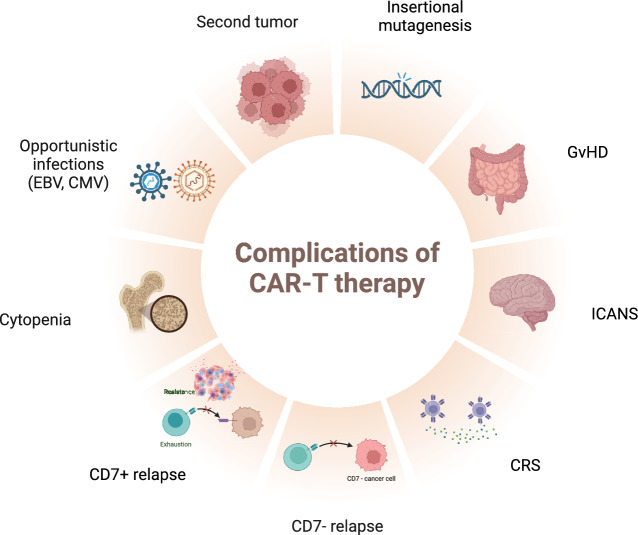
Complications of CAR-T cell therapy for T cell-derived malignancies. The common complications of various CAR-T cells include CRS, ICANS, GvHD, insertional mutagenesis, and second tumor. There are some complications that are specific for T-targeted CAR-T cell therapy, like opportunistic infections caused by T cel aplasia (such as EBV and CMV reactivation), persistent cytopenia caused by myelosuppression, and target antigen-negative relapse (such as CD7-negative relapse)

However, there are some specific adverse effects after T-targeted CAR-T cell therapy remain resolved, such as T cell aplasia and severe opportunistic infection Fig. 4. An Epstein-Barr virus (EBV)-associated second tumor was also observed in several studies: our group reported a patient diagnosed with EBV-associated diffuse large B cell lymphoma (DLBCL) on day 55 after CD7 CAR-T cell therapy, who eventually died of disease progression [13]; Chen et al. reported a case of EBV reactivation and post-transplant lymphoproliferative disease (PTLD) post-CD7 CAR-T cell infusion [23]; Two cases of EBV-associated PTLD were also reported in a CD5 CAR-T cell clinical study, and both died from treatment complications [9]. Disease relapse after CAR-T cell treatment is also a major problem. In the next paragraphs, we will introduce T cell immune reconstitution and disease relapse after CD5- or CD7-redirected CAR-T cell therapy.

### T cell immune reconstitution

T cell aplasia is a major challenge during the treatment of T cell-derived malignancies, which may cause uncontrolled opportunistic infections and threaten the lifespan. However, several studies have reported the expansion of CD7-negative T cells accompanied by the elimination of CD7-positive counterparts during CD7 CAR-T cell therapy [11, 13, 25]. Some studies found that these new occurring CD7-negative T cells showed an effector or effector memory phenotype with less naïve fraction and TCR diversity compared with T cells of healthy donors and CD19 CAR-T cell receivers [11, 26]. But Oh et al. demonstrated that the TCR diversity and T cell subsets in patients treated with CD7 CAR-T cells were comparable to those in patients treated with CD19 CAR-T cells [27]. In fact, there is a small population of naturally existing CD7-negative T cells among human all normal T cells (ranging from 90 to 96%), the percentage of which may be increased with age. These CD7-loss T cells predominantly presented the CD4 phenotype and the CD45RA-negative CD45RO-positive memory phenotype [11, 28, 29]. In some diseases, such as chronic viral infections (human immunodeficiency virus, EBV, etc.), rheumatoid arthritis, and kidney transplantation, the percentage of CD7-negative T cells increases significantly, which implies that the loss of the CD7 antigen may contribute to the terminal differentiation of repeatedly stimulated CD7-positive T cells [28]. A preclinical study reported that naturally existing CD7-negative T cells are capable of functional CD7-redirected fratricide-resistant CAR-T cell manufacturing, which was consistent with the conclusion of CAR-T cells knocking out of CD7, implying that CD7 may not be so critical in the maintenance of T cell function [30]. Though CD7 is an early T-committed marker, it may not contribute to T cell development either. Because CD7-KO human hematopoietic stem cells (HSCs) permitted normal T cell development and function. These CD7-KO HSC-derived T cells are closer to physiologically bulk T cells because they have a more naïve phenotype and proliferation ability than physiologically existing CD7-negative T cells [29]. However, whether CD7 really has a physiological role in T cell development and cytotoxicity, as well as the differences among CAR-T induced CD7-loss T cells, naturally existing CD7-negative T cells, and modified CD7-negative T cells, still needs more basic and clinical researches to explore.

To date, the immune function of these newly emerging CD7-negative T cells after CD7 CAR-T cell therapy remains controversial. Some studies believe that these CD7-loss T cells still perform a certain anti-infection function, but others believe that the cells are functionally deficient, which may cause viral reactivation (such as EBV and cytomegalovirus (CMV)) or other opportunistic infections after CAR-T cell treatment [11, 13]. Recently, single-cell RNA transcriptomic data revealed that emerging CD7-negative T cells expressed higher levels of T cell activation pathways than T cells before infusion, and monocyte reconstitution failure may account for severe infections (ChiCTR2200058969) [23].

In general, the origin and function of CD7-negative T cells are poorly understood. In the future, it will be necessary to explore whether CD7-loss T cells, especially newly emerging CD7-negative T cells after CD7 CAR-T cell treatment, remain resistant to opportunistic infections or how to improve the function of such T cells to improve patients' survival rates.

### Disease relapse

Several studies have reported disease recurrence on T-targeted CAR-T cell therapy [8, 11, 25]. It is divided into antigen-positive and antigen-negative relapses according to the presence or absence of surface target antigens. According to previous studies, the poor persistence/cytotoxicity of CAR-T cells and the negative regulation of tumor microenvironment may contribute to antigen-positive relapse. The potential mechanisms for antigen-negative relapse include antigen escape and the expansion of antigen-negative leukemic cells existed prior to infusion. The antigen escape can still be divided into malignant cell gene mutations, promoter hypermethylation, and alternative splicing, which may occur during CAR-T cell surveillance [31]. In a long-term follow-up study (ChiCTR2000034762), four of six relapsed patients were CD7-negative relapses, and next-generation sequencing showed two frameshift insertions and two missense mutations [25]. Gene mutations may explain CD7-negative relapses in these patients. However, due to the lack of pre-treatment sequencing data, whether these CD7-negative relapses originated from CD7-negative leukemic cells that were already present before treatment or from new mutations caused by treatment remained to be investigated [25]. Another article found that CD7-negative recurrences were contributed to CD7 transcriptional silencing rather than genetic mutations, although the exact source of these CD7-negative recurrences was also unclear [26].

One strategy for treating antigen-negative relapse is to change the CAR-T cell targets. In 2023, Pan et al. reported a phase I clinical trial (NCT05032599) in which donor-derived CD5-KO CD5-redirected CAR-T cell therapy was effective for CD7-negative relapsed T-ALL after CD7 CAR-T cell treatment. All 12 patients achieved CR within 1 month. However, double CAR-T cell infusions may increase the risk of viral and bacterial opportunistic infections in the long term, perhaps because of CD5 and CD7 double-negative T cells [12].

Relapse after CAR-T cell therapy may be prevented by bridging to HSCT. In 2023, Lu et al. reported that patients who received HSCT after CAR-T cell therapy achieved a higher progression-free survival rate than those who did not receive HSCT (67.2% versus 15.0%) [32]. The group also reported a parallel comparison between HSCT recipients who achieved CR with CD7 CAR-T cells or chemotherapy and found that either CAR-T cell therapy or chemotherapy-induced CR, followed by HSCT consolidation, can achieve good safety and efficacy [33]. The results illustrated that CD7 CAR-T cell therapy is an alternative strategy for achieving CR in patients who are chemotherapy-insensitive and gives these ineligible patients a chance for transplantation. Though sequential HSCT after CAR-T cell therapy can prevent disease relapse, acute GvHD is an inevitable complication of HSCT consolidation and an independent risk factor for OS rate [34].

Recently, our group reported a novel strategy for treating R/R acute leukemia by combining allogeneic CD7 CAR-T cell therapy and haploidentical HSCT. Of ten patients, nine received haploidentical CD7 CAR-T cells, while one received universal CD7 CAR-T cells. Under these conditions, subsequent HSCT does not require an additional myeloablative conditioning regimen or GvHD prophylaxis, which can prolong the persistence of CAR-T cells and reduce chemotherapy-related toxicities. In our clinical trial (NCT04599556 and NCT04538599), eight of nine evaluable patients presented complete chimerism after HSCT, and the other presented autologous hematopoietic recovery possibly due to the delayed infusion and compromised expansion of CAR-T cells. Only three patients experienced manageable acute GvHD (grade 2), and no chronic GvHD was observed. Six patients remained MRD-negative CR with a median follow-up time of 15.1 months. All eight patients that achieved complete donor chimerism had a CD7-negative phenotype in normal T cells. In addition, the remaining CD7-negative T cells maintained the ability to activate but with low allo-reactivity. All these data showed that long-lasting CAR-T cells after HSCT had a persistent killing ability of CD7-positive tumor cells and T cells contributing to prevention of leukemia relapse and GvHD. However, EBV and CMV reactivation occurred in almost all patients (nine of ten), and one patient was diagnosed with PTLD. Two patients relapsed with CD7-negative malignant cells, which can be explained by preexisting CD7-low or CD7-negative blasts [26]. This strategy of sequential CD7 CAR-T cell therapy and haploidentical HSCT offers a feasible approach for patients with CD7-positive tumors who are ineligible for conventional allogeneic HSCT. However, larger clinical cohort is needed for more substantial conclusions.

In conclusion, there is no effective strategy currently to prevent or treat disease recurrence, especially antigen-negative relapses. More deeper explorations on relapse mechanisms after T-targeted CAR-T cell therapy may help to address this problem.

## Future perspectives in CAR-T cell therapy for T cell-derived malignancies

CAR-T cell therapy has brought new hope for patients with T cell-derived malignancies. But the T-targeted CAR-T cell therapy is infant, and its safety and efficacy need more basic and clinical trials to confirm. There are special challenges in T-targeted CAR-T cells, such as fratricide, T cell aplasia, and tumor contamination [4]. We need to focus on these specific challenges while exploring general optimization strategies in the CAR-T cell modification and utilization to improve the efficacy of T-targeted CAR-T cells in clinic.

Improving the selection of the target antigens is an important strategy to solve the problem of fratricide and T cell aplasia. At present, the most widely used target antigens for T cell-derived malignancies are CD7 and CD5, both antigen-targeted CAR-T cells showed preliminary good safety and efficacy in clinical studies [5, 11]. Since CD7 and CD5 belong to pan-T targets, the fratricide and T cell aplasia are serious. Restricted-T antigens are expressed on tumor cells but only a group of normal T cells. Thus, these antigen-targeted CAR-T cells is a potential way to alleviate the fratricide and T cell aplasia. The clinical use of restrictive antigens like CD4 and TRBC1 were confined due to the low ratio of T cell-derived malignancies expressing restrictive antigens and killing by stimulated normal T cells [6, 7]. Other restrictive antigens such as CD1a, CD21, CD30, CD37, CCR4 and CCR9, are under preclinical and clinical studies [2]. Therefore, antigen screening for each patient who intends to receive CAR-T cell therapy will help them identify optimal antigens.

Although gene-edited universal CAR-T cells can address issues such as tumor contamination, GvHD, and allograft rejection, the risk of genotoxicity associated with the CRISPR/Cas9 system should not be overlooked [13, 14]. Base editing is regarded as a safer editing tool as it does not rely on double-stranded DNA breakage and could be an ideal gene-editing means to evade genotoxicity [15]. The short in vivo persistence remains a challenge for universal CAR-T cells. The recovered CD7-negative T cells after universal CD7 CAR-T cell therapy can resist the killing of CAR-T cells and play a role in the allo-rejection of CAR-T cells, which might contribute to the short duration of universal CD7 CAR-T cells in vivo [13]. We anticipate more approaches to extend the in vivo persistence of universal CAR-T cells for T cell-derived malignancies in the future to achieve better clinical outcomes.

Researchers have shown the clinical safety and efficacy of CD7 CAR-T cells that target CD7-positive cancers. However, there are disease progression or recurrence after CD7-targeted CAR-T cell treatment in clinic [8, 11, 25]. Dual-targeted CAR-T cells, such as CD5/CD7 CAR-T cells, may improve efficacy and reduce relapse after CAR-T cell therapy [35]. But these dual-targeted CAR-T cells are still under preclinical study, and we look forward to clinical trials of dual-targeted CAR-T cells to explore the safety and efficacy of this therapy. Bridging transplantation can effectively prevent relapse and rebuild the immune system after CAR-T cell therapy [32, 33]. Our new strategy of sequential CD7-targeted CAR-T cell therapy and HSCT without GvHD prophylaxis, which makes good use of the myelosuppression state after CAR-T cell infusion, is also suitable for patients with poor physical status [26].

## Conclusions

CAR-T cell therapy for T cell-derived malignancies is emerging and promising. Clinical studies have demonstrated the high safety and efficacy of this novel therapy; however, specific limitations exist, including fratricide, T cell aplasia, and tumor contamination. There are several ways to overcome fratricide, including artificial reduction of surface target antigens (gene-editing and ER and/or Golgi-retaining) and natural selection. Universal CAR-T cells may overcome several problems induced by autologous CAR-T cells, such as tumor contamination, poor-quality and delayed manufacturing. Currently, bridging to HSCT consolidation is recommended after CAR-T cell therapy, which may prevent relapse and rebuild the immune system. Our group reported a new strategy combining CD7 CAR-T cell therapy and sequential haploidentical HSCT without GvHD prophylaxis, providing an alternative approach for patients with CD7-positive malignancies who are ineligible for conventional allogeneic HSCT. Overall, CAR-T cell therapy is a novel and promising treatment for T cell-derived malignancies. We believe that T-targeted CAR-T cells will provide hope to patients in the future.

## Data Availability

No datasets were generated or analysed during the current study.

## Abbreviations

T-ALL: T cell acute lymphoblastic leukemia/lymphoma
PTCLs: Peripheral T cell lymphomas
CR: Complete remission
OS: Overall survival
HSCT: Hematopoietic stem cell transplantation
R/R: Relapsed/refractory
GvHD: Graft versus host disease
CAR: Chimeric antigen receptor
TCLs: T cell lymphomas
CPISPR: Clustered regularly interspaced short palindromic repeats
Cas9: CRISPR-associated protein 9
ER: Endoplasmic reticulum
FCM: Flow cytometry
qPCR: Quantitative polymerase chain reaction
TKIs: Tyrosine kinase inhibitors
MRD: Minimal residual disease
TRBC1: T cell receptor beta constant 1
TCR: T cell receptor
T-LBL: T cell lymphoblastic lymphoma
KO: Knockout
HLA: Human leukocyte antigen
AML: Acute myeloid leukemia
ZFN: Zinc finger nuclease
TALEN: Transcription activator-like effector nuclease
CRS: Cytokine response syndrome
ICANS: Immune effector cell-associated neurotoxicity syndrome
EBV: Epstein-Barr virus
DLBCL: Diffuse large B cell lymphoma
PTLD: Post-transplant lymphoproliferative disease
HSCs: Human hematopoietic stem cells
CMV: Cytomegalovirus
